# Enhanced Detection of Bacterial Ocular Pathogens: A Comparative Study of Broad-Range Real-Time PCR and Conventional Culture Methods

**DOI:** 10.3390/diagnostics15080966

**Published:** 2025-04-10

**Authors:** Sunggyun Park, Kyoungbo Kim, Youhyun Lee, Namhee Ryoo

**Affiliations:** 1 Departments of Laboratory Medicine, Keimyung University School of Medicine, Daegu 42601, Republic of Korea; nosdolu1@gmail.com (S.P.);; 2 Departments of Ophthalmology, Keimyung University School of Medicine, Daegu 42601, Republic of Korea

**Keywords:** ocular infections, real-time PCR, broad-range PCR

## Abstract

**Background**: Ocular infections can cause severe complications, including blindness, and distinguishing bacterial from fungal keratitis based on clinical features alone is difficult. This study compared broad-range conventional PCR and real-time PCR methods targeting the *16S rRNA* gene with traditional culture for diagnosing bacterial ocular infections. **Methods**: We analyzed 160 ocular specimens from 111 patients, categorizing them as septic or aseptic. The results of both conventional PCR and real-time PCR methods targeting the *16S rRNA* gene were compared with traditional culture outcomes. **Results**: Real-time PCR demonstrated higher sensitivity than conventional PCR, and receiver operating characteristic analysis determined optimal ΔCT cutoff values of −2.13 and −4.09 for septic and aseptic specimens, respectively. Delays in specimen processing significantly affected real-time PCR accuracy. The *16S rRNA* meta-taxonomic analysis using nanopore sequencing only validated the PCR results when the DNA concentration was sufficient. **Conclusions**: Broad-range real-time PCR proved to be a valuable diagnostic tool, particularly in aseptic specimens, with greater sensitivity and specificity than conventional PCR. The established ΔCT cutoff values improved diagnostic accuracy and showed that standardized specimen collection and processing are crucial for maximizing PCR efficacy.

## 1. Introduction

Ocular infection refers to various infectious diseases of the eye and is classified according to the affected anatomical structure as conjunctivitis, keratitis, orbital cellulitis, uveitis, retinitis, or endophthalmitis. These infections are of significant concern for both individuals and communities, due to complications leading to blindness and the associated socioeconomic impact [1].

The most common ocular infection is conjunctivitis. This inflammatory disease affects the conjunctival mucosa and imposes a significant economic and social burden [2]. The most severe ocular infection is keratitis, a leading cause of corneal blindness [3]. Exogenous endophthalmitis is an infectious complication that arises following cataract surgery, ocular surgery, or ocular trauma and is caused by the introduction of infectious pathogens such as bacteria. In contrast, endogenous endophthalmitis occurs due to the spread of pathogenic infections from elsewhere in the body.

If conjunctivitis or keratitis is not diagnosed and treated early, it poses a potential risk for other external or internal ocular infections [4,5], which, if left untreated, can damage ocular structures, leading to visual impairment and blindness. Tears contain antimicrobial substances that continuously protect the eye; however, once an injury or inflammation occurs, treatment is challenging and requires immediate management [6].

However, according to Dalmon et al., even corneal specialists correctly differentiate bacterial keratitis from fungal keratitis in less than 70% of cases, highlighting the difficulty of clinical diagnosis [7]. Therefore, to ensure the proper treatment of ocular infections, the causative organism must be accurately identified for rapid diagnosis and prompt treatment [3].

The development of several new technologies in recent years has reduced the turnaround time compared with traditional diagnostic approaches for identifying infectious pathogens [8]. DNA-based techniques primarily focus on rapid pathogen identification, and their applications are increasing across various fields. Specifically, numerous studies have evaluated the use of real-time PCR or microbiome analysis via next-generation sequencing (NGS) for their utility in ophthalmic disease diagnosis [9,10,11,12,13,14]. Nonetheless, culture remains the gold standard for pathogen identification in ocular infections. Since it often takes more than a day to identify the causative agent of ocular infections, an early screening method to confirm whether the infection is bacterial before pathogen identification takes place would be beneficial for initiating prompt treatment.

The ocular specimens typically encountered in clinical microbiology laboratories are mainly derived from cases of keratitis or endophthalmitis requiring culture tests. Specimens from cases of conjunctivitis are rare because this condition often resolves itself naturally or is empirically treated without culture tests. Most ocular specimens encountered in clinical laboratories are low in volume and are presumed to have a low microbial load [15,16]. Additionally, due to the anatomical structure of the eye, many septic samples often harbor normal commensal organisms as part of the ocular microbiota. These characteristics of ocular specimens affect not only the diagnostic performance of conventional microbiological tests, such as culture and Gram staining, but also the performance of various DNA-based techniques. PCR-based techniques, including conventional PCR, real-time PCR, and *16S rRNA*-targeted meta-taxonomic analysis, may be particularly advantageous for specimens with a low microbial load as they amplify and enrich the pathogen’s nucleic acids for detection [15,16]. Recently, as NGS-based sequencing and analysis have become possible with very small amounts of DNA, the utility of metagenomic analysis using shotgun sequencing has been reported [15,16,17].

Although culture is the current gold standard for diagnosing ocular infections, it has certain limitations, such as low sensitivity in small-volume or low-burden specimens and delayed turnaround time [8]. Molecular methods may offer faster and more sensitive detection, but their clinical utility in ocular specimens is not yet well established. Therefore, a direct comparison of molecular and culture-based methods is needed to assess their diagnostic value.

This study aimed to compare the diagnostic performance of molecular methods—including broad-range conventional PCR and real-time PCR targeting the *16S rRNA* gene—with that of traditional culture-based methods for identifying bacterial ocular infections. Additionally, we conducted *16S rRNA* meta-taxonomic analysis using nanopore sequencing to further assess the utility of molecular approaches. Although the primary focus was on bacterial pathogens, exploratory broad-range fungal PCR was also performed in a limited number of specimens.

## 2. Materials and Methods

### 2.1. Patients and Specimens

From April 2021 to January 2024, we performed conventional PCR and real-time PCR assays targeting the *16S rRNA* gene, using 160 residual ocular specimens from 111 patients. These specimens were originally submitted for culture tests to confirm ocular infections, but PCR was performed directly on the residual specimens by extracting nucleic acids, without prior culture, at a single institution in South Korea (Table 1). For further analysis, the ocular specimens were broadly classified as septic (samples typically expected to harbor bacteria, e.g., cornea, conjunctiva, contact lens, discharge, contact lens solution, and foreign body specimens) or aseptic (samples typically expected to be sterile, e.g., vitreous humor, aqueous humor, balanced salt solution, and intraocular lens specimens). This study was approved and the need to obtain informed consent was waived by the Institutional Review Board of Keimyung University’s Dongsan Medical Center (24 January 2023) and was conducted according to the principles of the Declaration of Helsinki.

### 2.2. Culture

As part of the routine culture-based diagnostic process, all 160 ocular specimens were first cultured, before the residual samples were used for PCR. For swab specimens (e.g., from the cornea, discharge, and conjunctiva) and lens and foreign body specimens, the samples were spread onto a blood agar plate and then placed in thioglycolate broth for culture at 36 °C for up to 48 h. Other specimen types (e.g., aqueous humor, vitreous humor, balanced salt solution, and contact lens solution) were also cultured on blood agar plates and in thioglycolate broth. In cases where additional fungal cultures were ordered, the specimens were cultured on Sabouraud dextrose agar at 36 °C for 48 h. If two or more swabs were available, or if the specimen volume was sufficient after performing the above culture tests, the residual specimens were placed in distilled water or thioglycolate broth to meet the minimum volume requirement (300 µL) for nucleic acid extraction and then stored at 4 °C for subsequent PCR analysis.

### 2.3. Nucleic Acid Extraction and PCR

For nucleic acid extraction, the refrigerated residual specimens were placed in distilled water or thioglycolate broth and processed using a QIAamp DSP DNA Mini Kit (Qiagen, Redwood City, CA, USA) with 300 µL of the corresponding medium. All procedures were performed per the manufacturer’s recommendations using an automated QIAcube system (Qiagen, Redwood City, CA, USA).

Conventional PCR for bacteria and fungus was performed using a total reaction volume of 16 µL, containing 3 µL of extracted DNA and 0.4 µM of each primer set. For real-time PCR for bacteria, the total reaction volume was 50 µL, containing 4 µL of extracted DNA, 0.5 µM of each primer set, and 0.25 µM of the probe. For real-time PCR for fungus, the total reaction volume was 50 µL, containing 5 µL of extracted DNA, 1.8 µM of each primer set, and 0.225 µM of the probe. Table 2 presents the PCR conditions and primer sets used for conventional PCR and real-time PCR. Each PCR run included negative controls (PCR-grade distilled water) and positive controls (DNA extracted from a standard *Escherichia coli* strain and a clinical isolate of *Fusarium oxysporum*).

**Figure 1:**
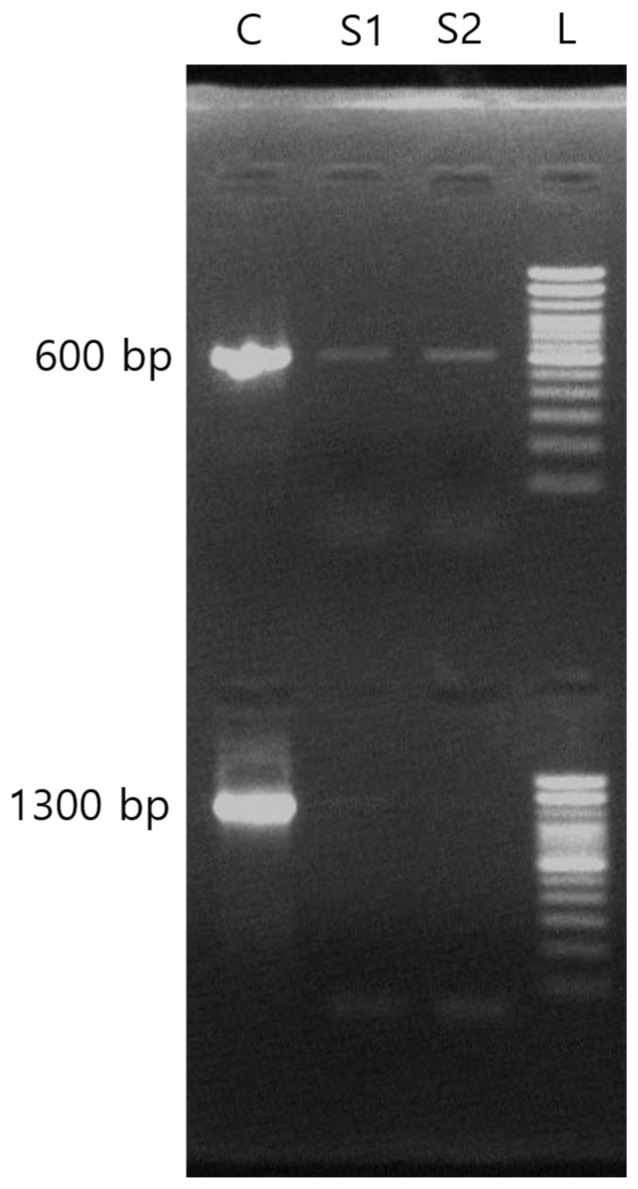
Agarose gel electrophoresis of conventional PCR products. Lane L represents the size ladder; lane C represents the positive control; lanes S1 and S2 represent ocular specimens 1 and 2, respectively.

For conventional PCR, a positive result was determined by the visual confirmation of an amplified band of approximately 1300 bp for bacteria and 600 bp for fungus following electrophoresis (Figure 1).

For real-time PCR for bacteria, the result was assessed using the ΔCT value, calculated as follows: (CT value of the sample) − (CT value of the negative control), as all negative controls showed amplification. The cutoff for the ΔCT value was determined using receiver operating characteristic (ROC) curve analysis, based on ΔCT values corresponding to positive culture results (see Section 3.2). Accordingly, ΔCT values of <−4.09 for aseptic specimens and <−2.13 for septic specimens were considered positive. For real-time PCR targeting fungi, a positive result was determined if amplification was observed within 50 cycles.

To determine the clinical significance of *16S rRNA*-targeted broad-range real-time PCR in excluding fungal ocular infections, we conducted broad-range conventional PCR and real-time PCR targeting fungal sequences. We examined the difference in positivity rates of real-time PCR for fungi, based on the positive or negative results of ΔCT values from real-time PCR for bacteria. This analysis was performed on 151 specimens (excluding 4 septic and 5 aseptic specimens on which real-time PCR targeting fungi was not performed), which underwent both real-time PCR targeting fungal 18S rRNA and conventional PCR targeting the D1/D2 region.

### 2.4. Validation of an Appropriate Solution for Ocular Specimens in Nucleic Acid Extraction for Identifying Bacteria

In our institution, ocular specimens are routinely collected in thioglycolate broth to allow for optional culture testing. However, since thioglycolate broth contains organic components, we sought to confirm whether its use could affect nucleic acid extraction efficiency compared to commonly used inert solutions, such as saline or distilled water. Therefore, this comparative experiment was conducted to assess whether the use of thioglycolate broth influences the quality of extracted bacterial DNA.

To evaluate this, we compared the ΔCT values of 15 culture-positive specimens among 37 ocular specimens collected between April 2021 and March 2023. These specimens were of sufficient volume to be divided into both distilled water and thioglycolate broth groups. A paired *t*-test was performed on the ΔCT values for each matched pair, and *p*-values of <0.05 were considered statistically significant.

### 2.5. Analysis of the Causes of Discrepancies Between Bacterial Culture Tests and Bacterial Real-Time PCR Results

We investigated those factors that might reduce the accuracy of bacterial real-time PCR, thereby contributing to discrepancies between bacterial culture tests and bacterial real-time PCR results. After grouping the samples into those where the bacterial culture results matched the real-time PCR results and those where they did not, we analyzed differences in the mean or proportion of various factors between the two groups, including sex, age, the presence of diabetes, association with contact lens use, a history of ocular trauma, a history of invasive ophthalmic procedures or surgeries within 1 year of specimen collection, whether the patient required hospitalization (an indicator of symptom severity), Gram stain results, and the time elapsed from specimen collection to nucleic acid extraction for PCR.

All statistical analyses were performed using R v4.3.1 software. We used Student’s *t*-test and the Mann–Whitney U test to compare the means of continuous variables between groups. To compare the proportions of categorical variables between the groups, we used Pearson’s chi-square test and Fisher’s exact test.

### 2.6. 16S rRNA Meta-Taxonomic Analysis

To verify the reliability of the bacterial real-time PCR results, we performed *16S rRNA* meta-taxonomic analysis on 21 low-burden specimens that were deemed positive based on the bacterial real-time PCR results (ΔCT values below the cutoff) but that did not show growth in cultures, along with 14 high-burden specimens that tested positive by both real-time PCR (ΔCT values below the cutoff) and the culture. Briefly, the PCR amplicons were prepared for NGS using the Oxford Nanopore Ligation sequencing amplicon-native barcoding protocol (SQK-LSK109 with EXP-NBD104). To ensure test reliability, we used amplicons from conventional PCR for bacteria with a longer amplicon length. Sequencing was performed using a MinION nanopore sequencing device (Oxford Nanopore Technologies, Oxford, UK). To ensure reliable sequencing, the nucleic acid concentration of the amplicon was set at a minimum of 100 fmol (at least 1.76 ng/µL for a 1.3 kb amplicon) in a maximum volume of 48 µL. If these conditions were not met, this implied that the results would be unreliable, and such amplicons did not undergo *16S rRNA* meta-taxonomic analysis. DNA concentration was measured using a Qubit fluorometer (Thermo Fisher Scientific, Waltham, MA, USA).

## 3. Results

### 3.1. Validation of the Selection of an Appropriate Solution for Ocular Specimens in Nucleic Acid Extraction

**Figure 2:**
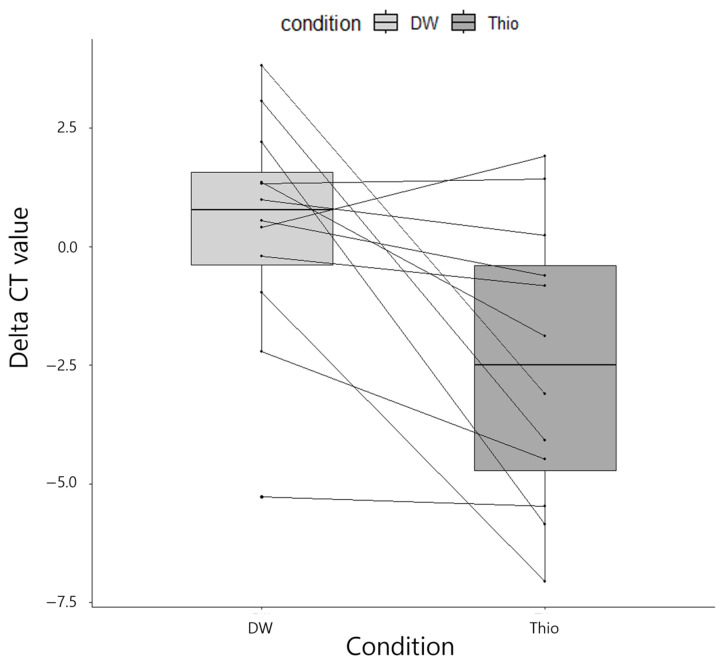
Comparison of ΔCT values of the ocular samples stored in different solutions. The thioglycolate-stored specimens had a significantly lower ΔCT value compared with the specimens stored in distilled water (*p* = 0.01105). Abbreviations: DW, distilled water; Thio, thioglycolate broth.

A comparison of ΔCT values among the 15 specimens that were positive for culture and that had both samples placed in thioglycolate and distilled water revealed that the thioglycolate-placed specimens had a significantly lower ΔCT value (by approximately 2.91) compared with the distilled water-placed specimens (*p* = 0.01105; Figure 2). Therefore, we decided to use thioglycolate as the solution to directly process the ocular specimens for nucleic acid extraction in subsequent analyses.

### 3.2. Setting the ΔCT Cutoff Value for Bacterial Real-Time PCR Results Interpretation

**Figure 3:**
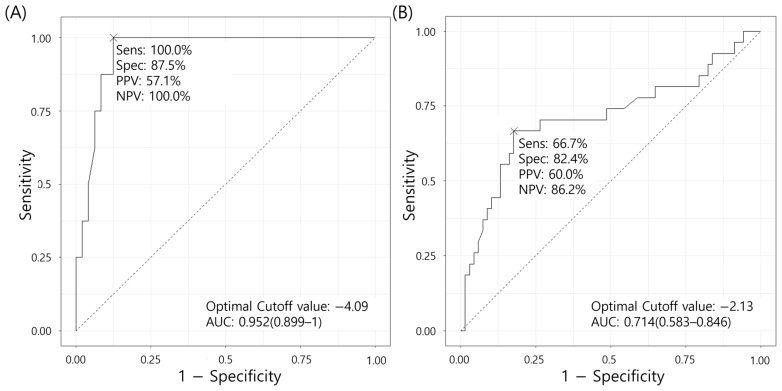
ROC curve analysis of ΔCT values. (**A**) Aseptic specimens and (**B**) septic specimens, compared with the culture results of occasional or more than occasional colonies. Abbreviations: Sens, sensitivity; Spec, specificity; PPV, positive predictive value; NPV, negative predictive value; AUC, area under the curve.

Analysis was conducted separately for septic (*n* = 101) and aseptic (*n* = 59) specimens. We compared the measured ΔCT values with the culture outcomes to determine the ΔCT value cutoff that best predicted the culture results. To minimize the possibility of bacterial culture contamination, we performed ROC curve analysis by categorizing the positive cases into two groups: those with ≥10 colonies in the first quadrant of the plate (occasional or more than occasional colonies) (Figure 3) and those with any positive result, including specimens with a single colony (rare colonies or those with no growth) (Supplementary Materials, Figure S1). The area under the curve (AUC) was higher when only those cases with occasional or more than occasional colonies were considered positive, with a ΔCT cutoff value of −2.13 for septic specimens and −4.09 for aseptic specimens. The AUC was 0.714 (95% confidence interval: 0.583–0.846) for septic specimens and 0.952 (0.899–1) for aseptic specimens, indicating a higher accuracy for the aseptic specimens.

### 3.3. Bacterial Conventional PCR Versus Real-Time PCR

We analyzed the results of conventional PCR and real-time PCR, both targeting the *16S rRNA* gene with different primers, and compared them with the culture results (Table 3 and Table S1). For septic specimens, bacterial real-time PCR achieved a sensitivity of 64.3%, significantly outperforming conventional PCR performance at 42.9%. The difference was even more pronounced in aseptic specimens, where bacterial real-time PCR demonstrated an impressive sensitivity of 88.9%, in stark contrast with conventional PCR sensitivity at 33.3%. The specificity of bacterial real-time PCR was 83.6% for septic specimens and 88% for aseptic specimens, while conventional PCR showed specificities of 79.5% and 96%, respectively, indicating similar levels. A comparison of the results of bacterial real-time PCR with conventional PCR revealed an overall concordance of approximately 70% for both septic and aseptic specimens (Table 4).

### 3.4. Analysis of Causes of False-Negative Bacterial Real-Time PCR Results

**Figure 4:**
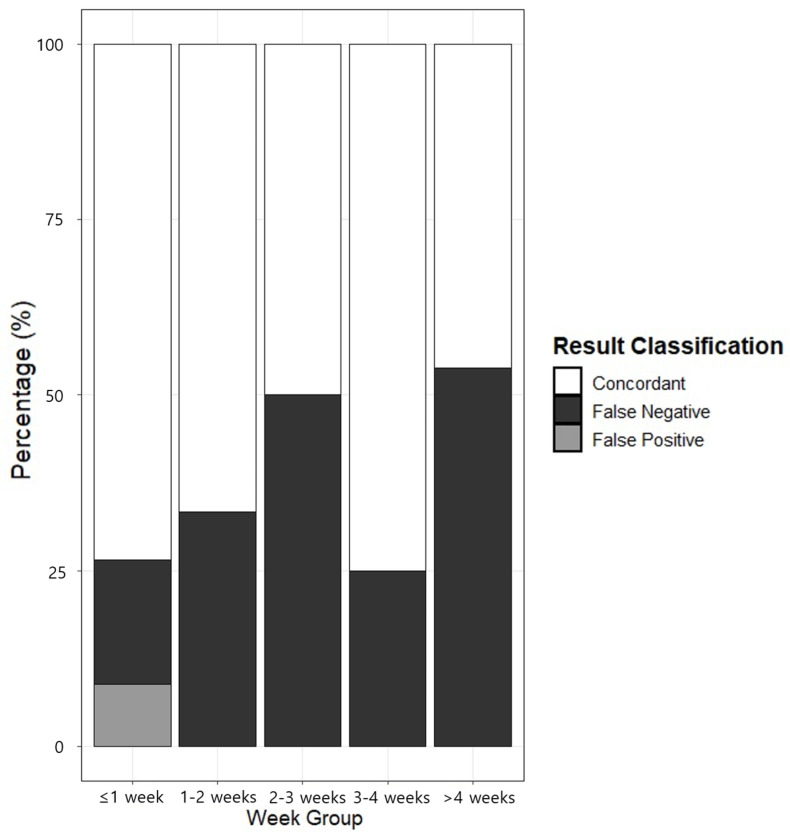
Concordance of real-time PCR results, shown by the time interval between sample collection and nucleic acid extraction.

An analysis of the factors affecting the positive concordance rate, in which both the bacterial culture results and the bacterial real-time PCR results were positive, revealed that the only statistically significant factor in septic specimens was the elapsed time from specimen collection to nucleic acid extraction for PCR (*p* = 0.003; Table 5). The concordance between the *16S rRNA* broad-range real-time PCR results and the culture results for the 160 specimens included in the study, along with the distribution of the elapsed time from specimen collection to nucleic acid extraction, is shown in Figure 4. There were no statistically significant factors that were identified under the same conditions for aseptic specimens (Table S2).

### 3.5. 16S rRNA Meta-Taxonomic Analysis

For the 21 low-burden specimens, where the bacterial real-time PCR results showed ΔCT values near or below the cutoff value (indicating a possible positive result), but where the bacterial culture results showed no growth, the DNA concentrations were too low to conduct reliable *16S rRNA* meta-taxonomic analysis (Table S5). Among the 14 high-burden specimens, where the bacterial real-time PCR results showed ΔCT values below the cutoff and the bacterial culture results were also positive, 10 specimens did not meet the required DNA concentrations. Thus, only the four remaining specimens underwent *16S rRNA* meta-taxonomic analysis using NGS, yielding results that were consistent with the culture outcomes (Table 6).

### 3.6. Broad-Range Fungal PCR and Bacterial Real-Time PCR

Out of 160 thioglycolate specimens, excluding 4 septic and 5 aseptic specimens where broad-range fungal PCR could not be performed, 5 out of 151 specimens were positive for fungal culture. These comprised three specimens of *Candida albicans* and two specimens of *Fusarium solani* (Supplementary Materials, Table S1). All five fungal culture-positive specimens were confirmed as positive by conventional PCR targeting the D1/D2 region. However, in the real-time PCR targeting 18S rRNA, only the two *Fusarium solani* specimens were confirmed positive. To examine the association between bacterial PCR and fungal PCR results, we compared the frequency of positive and negative fungal PCR results in groups with positive and negative ΔCT values from bacterial real-time PCR. Although not statistically significant, we observed that the fungal PCR positivity rate was lower in the bacterial ΔCT-positive group (9.3%) compared to the ΔCT-negative group (34.0%) in the septic ocular specimen (*n* = 97) (see Figures S2 and S3).

## 4. Discussion

Although DNA-based molecular techniques cannot entirely replace traditional culture-based microbiological testing, they offer a highly promising approach for rapid pathogen identification, enabling quicker diagnosis of infectious diseases [8]. In particular, broad-range PCR targeting the *16S rRNA* gene, using primers specifically designed to detect most bacteria, enables identification using sequence analysis of the amplified PCR product. The clinical utility of this technique has been widely reported in blood specimens, where polymicrobial infections are rare [19,20].

The rapid diagnosis of ocular bacterial infections is crucial for effective treatment. However, the current standard diagnostic methods of staining and culture are time-consuming and often fail to provide clinically useful results. This low sensitivity is mainly attributed to prior antibiotic use and the technical difficulties encountered when culturing microorganisms from small specimen volumes [21]. Thus, there have been numerous attempts to develop highly sensitive and accurate molecular diagnostic tools that can rapidly diagnose ocular infections [9,10,11,12,13,14]. However, distinguishing the pathogens from normal polymicrobial flora in certain ocular specimens remains a challenge, limiting the effectiveness of these molecular approaches.

Moreover, in this study, the results of our broad-range real-time PCR for bacteria showed the presence of peaks in the negative control, which comprised PCR-grade distilled water. Most of these CT values were >30, but some were <30 (Supplementary Materials, Table S1). This outcome can be attributed to contamination by ubiquitous microbial DNA, potentially originating from the PCR reagents, nucleic acid extraction kits, or even the molecular-grade water itself [22]. Such DNA contamination significantly impacts experiments, particularly those that are testing specimens with a low microbial load. Therefore, in this study, we assumed that the amount of pathogen DNA present in the specimens from patients with ocular infections would be greater than that of any microbial DNA contaminants. Thus, we determined the presence of pathogenic bacteria by calculating the difference between the CT value of the specimen and the CT value of the negative control in each run, referred to as the ΔCT value. By using the ΔCT value, we were able to account for the presence of pre-existing bacterial contaminants. However, since amplification was observed in all negative controls, we could not entirely exclude the possibility of cross-contamination between specimens during sample processing or PCR setup. This remains a limitation of our study. Nevertheless, considering that all experiments were conducted in a level 2 biosafety cabinet and were performed in a clinical laboratory where specimen handling and environmental contamination were carefully controlled, this limitation may be mitigated.

To select the appropriate solution for ocular specimens to nucleic acid extraction for bacterial real-time PCR, we compared the ΔCT values of specimens in distilled water with those in thioglycolate broth. The specimens in thioglycolate broth showed significantly lower ΔCT values compared with the specimens in distilled water. When extracting nucleic acid from small amounts of specimens or swab specimens, adding a specific solution to obtain the necessary volume for extraction is often necessary. Our results indicated that in such cases, the use of thioglycolate broth as the solution was more advantageous for bacterial detection.

Although thioglycolate contains organic nutrients that may promote bacterial growth, this could be advantageous in ocular specimens, which typically have low microbial loads. In such cases, limited bacterial proliferation may improve detection sensitivity. Since the overgrowth of commensal flora can be a concern with septic samples, we attempted to minimize this effect by applying ΔCT cutoff values to distinguish true pathogens from background signals.

The results of our ROC curve analysis, conducted to determine the ΔCT cutoff value, revealed that the AUC was higher in aseptic specimens than in septic specimens. This suggests that broad-range bacterial real-time PCR has greater diagnostic value in aseptic specimens, compared with septic specimens in which commensal bacteria are present.

When the results were determined using the established cutoff value for aseptic and septic specimens, respectively, both the sensitivity and specificity of bacterial real-time PCR compared with the bacterial culture results were higher than those of conventional PCR. However, a comparison of the results of bacterial real-time PCR with conventional PCR revealed a concordance of approximately 70%. Notably, bacterial real-time PCR was negative, while conventional PCR was positive in approximately 10% of cases (Supplementary Materials, Table S1). Its efficacy in detecting different bacteria varies, depending on the type of broad-range PCR primers used [19]. We only used one type of set of bacterial real-time PCR primers in our study, but using two or more primer sets (in multiplex real-time PCR) may potentially achieve higher sensitivity.

Nevertheless, in this study, the sensitivity of bacterial real-time PCR methods for septic specimens was 64.3%. If bacterial real-time PCR were used as an initial screening method, followed by culture testing for confirmation, false-negative results could hinder efficient testing. We identified the time that elapsed from specimen collection to nucleic acid extraction as the primary cause of these false-negative results, and the longer the time between collection and extraction, the more likely it was for false-negative results to occur in bacterial real-time PCR assays of culture-positive specimens. Thus, to achieve higher detection rates, it may be beneficial to select and use an appropriate transport medium, and this selection would require validation.

Recent studies have shown promising results using *16S rRNA* meta-taxonomic analysis or shotgun metagenomic analysis in ophthalmology [15,16,17]. In particular, shotgun metagenomic analysis has been reported to provide more comprehensive information, offering significant improvements in the diagnosis of ocular infections [15,16]. However, it is important to note that while these advanced sequencing techniques offer numerous advantages, they also present challenges. Economic cost issues, the time required for data analysis, and persistent problems with test standardization are obstacles to routine use in clinical laboratories that need to be overcome [15,16]. In this study, we performed a meta-taxonomic analysis targeting *16S rRNA* using a nanopore sequencing system. While this method is useful for analyzing bacterial communities in specimens with a low microbial load, it provides limited information compared to shotgun metagenomic sequencing, which can simultaneously obtain information on various pathogens, including bacteria, fungi, and viruses [15,16,17].

We aimed to validate our bacterial real-time PCR results using this *16S rRNA* meta-taxonomic analysis. However, all low-burden specimens with ΔCT values close to the cutoff did not meet the DNA quantity specifications for our protocol for *16S rRNA* meta-taxonomic analysis, and over half of the high-burden specimens with ΔCT values below the cutoff also failed to meet these conditions. Sagerfors et al. evaluated the utility of *16S rRNA* meta-taxonomic analysis in corneal diseases, reporting that approximately 83% of specimens were suitable for NGS analysis [14], which was higher than the analysis rate observed in our study. Although this difference may be attributed to various factors, a significant limitation of our study was that it was conducted using residual specimens. Unlike previous studies, which employed a standardized corneal scraping method and the same liquid transport medium, our study did not use standardized specimen collection methods. Furthermore, the Oxford nanopore ligation sequencing amplicons-native barcoding protocol (SQK-LSK109 with EXP-NBD104) used in this study is a simplified preparation protocol for amplicons, but it does require a relatively high DNA input amount. Additional research using protocols that require lower DNA input amounts for shotgun metagenome analysis is needed to more clearly validate the clinical effectiveness of ΔCT. Despite the limitations, in the four specimens where *16S rRNA* meta-taxonomic analysis was feasible, the results aligned with the culture findings. While this small sample size precludes definitive conclusions, it provides some preliminary support for the potential reliability of bacterial real-time PCR in these cases.

Following conjunctivitis, which is predominantly viral, keratitis is the second most common ocular infection, accounting for approximately 25–50% of all ocular infections, with 65–90% of them being bacterial in nature [23,24]. Additionally, distinguishing between bacterial and fungal keratitis based on the clinical features alone is known to be challenging [7]. Consequently, in most cases of keratitis, unless severe keratitis is suspected, empirical antibiotic treatment is initiated without specific microbiological testing [25]. If there is no response to initial empirical antibiotic therapy in about 20–30% of cases, microbiological tests are performed to identify other pathogens such as fungi [26]. However, it has been reported that many cases of keratitis involve polymicrobial infections, meaning that even when a fungal infection is confirmed, antibiotic treatment is often maintained in most cases [27,28]. If fungal infections can be ruled out quickly, unnecessary antifungal treatment can be avoided. Moreover, excluding the possibility of fungal infection is essential for the safe initiation of steroid therapy, and the performance of the existing time-consuming culture-based tests is inadequate for this decision. The results of our study, showing a lower frequency of positive fungal PCR in cases where bacterial real-time PCR was negative compared to positive cases, demonstrate that bacterial real-time PCR could be useful in reducing unnecessary antifungal treatments and enabling the prompt initiation of appropriate steroid therapy. However, while we performed broad-range fungal PCR assays in this study, we were unable to conduct shotgun metagenomic analysis, which precluded the accurate identification of fungal ocular infections. In addition, although fungal PCR was performed in a limited number of specimens, enzymatic lysis methods, such as lyticase treatment, were not employed. This may have reduced the efficiency of fungal DNA extraction, particularly given the difficulty of disrupting fungal cell walls using standard detergent-based methods. Furthermore, only a small number of fungal-infected ocular specimens were included, an amount that we considered insufficient for a reliable evaluation of the diagnostic performance of fungal-targeted molecular assays. Therefore, future studies using optimized fungal DNA extraction protocols and shotgun metagenomic analysis are warranted to better assess the clinical utility of fungal PCR and to further validate the performance of bacterial broad-range real-time PCR as a tool to support the early differential diagnosis of bacterial versus fungal ocular infections.

The strengths of this study lie in the use of a small sample volume to demonstrate the considerable performance of real-time PCR in diagnosing ocular bacterial infections from various sample types. While numerous studies have demonstrated the utility of broad-range real-time PCR in specimens with a presumably low bacterial burden, our study uniquely attempted to establish ΔCT cut-offs in non-sterile specimens, which we believe is a significant contribution to the diagnosis of ocular infections. Studies utilizing ΔCT values for diagnosing bacterial keratitis or endophthalmitis are rare. Shimizu et al. (2019) reported using real-time PCR to quantify *16S rRNA* copies, establishing a definitive clinical diagnostic cut-off of 6.9 × 10^4^ copies [29]. However, quantifying copy numbers via real-time PCR requires generating a standard curve, a process that demands excessive time and cost for its routine implementation in clinical laboratories. An approach using ΔCT could potentially overcome these limitations. Moreover, it may offer advantages in addressing contamination from ubiquitous microbial DNA. This method suggests the potential of using ΔCT as a biomarker for diagnosing bacterial infections, not only in ocular samples but also in other specimen types where only a small amount of pathogen is expected.

Our study has several limitations. First, we only used one set of bacterial real-time PCR primers, so we were unable to verify the presence of pathogens that were not detected due to sequence differences in primer binding sites. Additionally, our study was conducted retrospectively, using residual specimens, so we were unable to standardize and validate the specimen collection methods. Furthermore, as mentioned earlier, due to our study design, we could not completely rule out the possibility of cross-contamination between specimens during sample processing or PCR setup.

Another limitation of our study is that different bacterial species harbor varying numbers of *16S rRNA* gene copies, which we did not account for in our analysis. This variation could have influenced our interpretation of the bacterial loads in the specimens. Additionally, our study did not reflect the actual clinical impact of molecular analysis for diagnosing bacterial ocular infections, such as its influence on treatment selection or patient outcomes. In future studies, approaches that account for variations in *16S rRNA* gene copy number among different bacterial species and strategies to further minimize the risk of cross-contamination should be implemented to improve the accuracy and reliability of molecular diagnostics for ocular infections.

A major limitation of our study was our attempt to analyze microbial communities using only *16S rRNA* meta-taxonomic analysis. As mentioned earlier, while this method is effective for bacterial community analysis, it is limited in terms of detecting other microorganisms such as fungi and viruses. Moreover, the protocol that we used required a high DNA input, which significantly hindered our ability to fully evaluate the performance of *16S rRNA* broad-range real-time PCR. This limitation affected our assessment of its capability to diagnose bacterial ocular infections and exclude fungal ocular infections. In future studies, to overcome these limitations, shotgun metagenomic analysis using protocols with lower DNA input requirements should be considered.

However, it must be emphasized that we were able to validate the utility of real-time PCR using a wider variety of ocular specimens compared with previous studies. Furthermore, we attempted to apply broad-range PCR cutoff values in septic specimens using ΔCT values. We also sought to propose solutions to overcome the technique’s low sensitivity and aimed to evaluate its performance and clinical significance from various perspectives. Nevertheless, it is important to acknowledge that traditional culture remains the gold standard in microbiological diagnosis. While molecular methods offer significant advantages in terms of rapid detection, they do not provide the viable isolates required for antimicrobial susceptibility testing, which is essential for guiding appropriate therapy. Therefore, real-time PCR should be considered to be a complementary tool rather than a replacement for conventional culture, particularly in the clinical decision-making process regarding ocular infections.

In this study, we confirmed the utility of broad-range real-time PCR for screening and diagnosing bacterial ocular infections. Compared with septic specimens, we observed its higher sensitivity and specificity in aseptic specimens, such as with those tests used for diagnosing endophthalmitis. However, we also validated the diagnostic utility of real-time PCR in septic specimens, such as those samples taken from cases of keratitis. Improving sensitivity in septic specimens may be achieved by standardizing specimen collection methods, the use of appropriate transport media, and rapid nucleic acid extraction after specimen collection.

## Figures and Tables

**Table 1:** Descriptive statistics for the 111 patients and 160 specimens included in this study.

Patients	Variable	Number	%	Specimens	Variable	Number	%
Total		111	100	Total		160	100
Sex	M	64	57.66	Type	Cornea	78	48.75
	F	47	42.34		Anterior chamber	31	19.38
DM	Yes	32	28.83		Vitreous humor	23	14.38
	No	79	71.17		Contact lens	14	8.75
Clinical diagnosis	Keratitis	66	59.46		Discharge	4	2.50
	Endophthalmitis	32	28.83		Balanced salt solution	3	1.88
	Uveitis	4	3.60		Therapeutic contact lens	2	1.25
	Orbital/eyelid mass	2	1.80		Intraocular lens	2	1.25
	Orbital cellulitis	2	1.80		Contact lens solution	1	0.63
	Retinal vasculitis	1	0.90		Foreign body	1	0.63
	Scleritis	1	0.90		Conjunctiva	1	0.63
	Eyeball rupture	1	0.90	Culture (Septic, 101)	No growth	51	50.5
	Intraorbital foreign body	1	0.90		*Corynebacterium macginleyi*	9	8.91
	Conjunctivitis	1	0.90		*Staphylococcus epidermidis*	7	6.93
					*Staphylococcus aureus*	6	5.94
					*Serratia marcescens*	4	3.96
					*Pseudomonas aeruginosa*	3	2.97
					Others	21	20.79
				Culture (Aseptic, 59)	No growth	46	77.97
					*Pseudomonas aeruginosa*	5	8.47
					*Streptococcus mitis/oralis*	3	5.08
					*Corynebacterium striatum*	2	3.39
					*Streptococcus dysgalactiae*	2	3.39
					*Staphylococcus epidermidis*	1	1.69

**Table 2:** The oligonucleotides used as the primers and probes to identify part of the bacterial *16S rRNA* gene.

	Conventional PCR [13]	Real-Time PCR [18,19]
Target	*16* *S* *rRNA*	*16* *S* *rRNA*
Forward	5′-CAGGCCTAACAGATGCAAGTC-3′	5′-AGTTTGATCMTGGCTCAG-3′
Reverse	5′-GGGCGGWGTGTACAAGGC-3′	5′-GGACTACHAGGGTATCTAAT-3′
Probe	None	5′(HEX)-CGTATTACCGCGGCTGCTGGCAC-(BHQ1)3′
PCR conditions	Initial activation: 95 × 15 min Total cycles: 30 cycles Denaturation: 95 × 1 min Annealing: 55 × 1 min Extension: 72 × 1.5 min	Initial activation: 95 × 10 min Total cycles: 45 cycles Denaturation: 95 × 1 min Annealing: 50 × 1 min Extension: 72 × 1 min
Target	D1/D2 region	*18s rRNA*
Forward	5′-GCATATCAATAAGCGGAGGAAAAG-3′	5′-GGRAAACTCACCAGGTCCAG-3′
Reverse	5′-GGTCCGTGTTTCAAGACG-3′	5′-GSWCTATCCCCAKCACGA-3′
Probe	None	5′(FAM)-TGGTGCATGGCCGTT-(BHQ1)3′
PCR conditions	Initial activation: 95 × 15 min Total cycles: 35 cycles Denaturation: 95 × 30 s Annealing: 50 × 30 s Extension: 72 × 30 s	Initial activation: 95 × 10 min Total cycles: 50 cycles Denaturation: 95 × 15 s Annealing & extension: 65 × 1 min

**Table 3:** Performance of bacterial conventional PCR and real-time PCR compared with the culture results.

Specimen Type (No.)	Growth over Occasional		Growth Under Occasional or No Growth		Sensitivity (95% CI)	Specificity (95% CI)
	No. of Positive Real-Time PCR (%)	No. of Negative Real-Time PCR (%)	No. of Positive Real-Time PCR (%)	No. of Negative Real-Time PCR (%)		
Septic (101)	18 (48.6)	10 (27.0)	12 (9.8)	61 (49.6)	0.643 (0.441–0.814)	0.836 (0.73–0.912)
Aseptic (59)	8 (21.6)	1 (2.7)	6 (4.9)	44 (35.8)	0.889 (0.518–0.997)	0.88 (0.757–0.955)
Specimen type (No.)	Growth over occasional		Growth under occasional or no growth		Sensitivity (95% CI)	Specificity (95% CI)
	No. of positives with conventional PCR (%)	No. of negatives with conventional PCR (%)	No. of positives with conventional PCR (%)	No. of negatives with conventional PCR (%)		
Septic (101)	12 (32.4)	16 (43.2)	15 (12.2)	58 (47.2)	0.429 (0.245–0.628)	0.795 (0.684–0.88)
Aseptic (59)	3 (8.1)	6 (16.2)	2 (1.6)	48 (39.0)	0.333 (0.075–0.701)	0.96 (0.863–0.995)

**Table 4:** Concordance between bacterial conventional PCR and real-time PCR.

Specimen Type (No.)	Positive for Real-Time PCR		Negative for Real-Time PCR		Agreement (95% CI)
	No. of Positives with Conventional PCR (%)	No. of Negatives with Conventional PCR (%)	No. of Positives with Conventional PCR (%)	No. of Negatives with Conventional PCR (%)	
Septic (101)	14 (43.8)	13 (40.6)	16 (12.5)	58 (45.3)	0.713 (0.618–0.792)
Aseptic (59)	2 (6.3)	3 (9.4)	12 (9.4)	42 (32.8)	0.746 (0.622–0.839)

**Table 5:** Variables affecting the positive concordance between bacterial culture and real-time PCR in septic specimens.

Variable		Growth on Culture		*p*-Value
		Negatives with Real-Time PCR (*N* = 29)	Positives with Real-Time PCR (*N* = 11)	
Sex	Female	18 (62.1%)	5 (45.5%)	0.477
	Male	11 (37.9%)	6 (54.5%)	
Age		47 ± 21.5	43.3 ± 21.1	0.629
DM	No	17 (58.6%)	9 (81.8%)	0.27
	Yes	12 (41.4%)	2 (18.2%)	
Contact lens	No	20 (69.0%)	7 (63.6%)	1
	Yes	9 (31.0%)	4 (36.4%)	
Trauma	No	29 (100.0%)	11 (100.0%)	NA
Operation history	No	20 (69.0%)	6 (54.5%)	0.469
	Yes	9 (31.0%)	5 (45.4%)	
Admission	No	16 (55.2%)	4 (36.4%)	0.479
	Yes	13 (44.8%)	7 (63.6%)	
Gram stain	Negative	16 (55.2%)	2 (18.2%)	0.073
	Positive	13 (44.8%)	9 (81.8%)	
Sample to extraction (day)		10 (0–113)	1 (0–15)	0.003

Abbreviations: DM, diabetes melitus; NA, not applicable.

**Table 6:** Metataxonomic analysis results of four ocular specimens.

No.	Clinical Diagnosis	Specimen	ΔCT	DNA Concentration (ng/µL)	Culture Results	Microbiome Results
17	Endophthalmitis	Intraocular lens	−10.41	37.45	*Pseudomonas aeruginosa*, occasional	*Pseudomonas aeruginosa*
117	Keratitis	Contact lens	−8.16	11.2	*Serratia marcescens*, many *Bacillus cereus*, occasional	*Serratia marcescens*
15	Endophthalmitis	Cornea	−5.15	19.95	*Pseudomonas aeruginosa*, moderate	*Pseudomonas aeruginosa*
20	Keratitis	Contact lens	−7.27	46	*Aeromonas hydrophila*, many	*Aeromonas hydrophila*
No.	Clinical diagnosis	Specimen	ΔCT	DNA concentration (ng/µL)	Culture results	Microbiome results
17	Endophthalmitis	Intraocular lens	−10.41	37.45	*Pseudomonas aeruginosa*, occasional	*Pseudomonas aeruginosa*
117	Keratitis	Contact lens	−8.16	11.2	*Serratia marcescens*, many *Bacillus cereus*, occasional	*Serratia marcescens*
15	Endophthalmitis	Cornea	−5.15	19.95	*Pseudomonas aeruginosa*, moderate	*Pseudomonas aeruginosa*
20	Keratitis	Contact lens	−7.27	46	*Aeromonas hydrophila*, many	*Aeromonas hydrophila*

## Data Availability

The datasets used and analyzed during the current study are available from the corresponding author upon reasonable request. Raw sequencing data generated during the current study are available in the BioProject repository (PRJNA1175405).

## Supplementary Materials

The following supporting information can be downloaded at: https://www.mdpi.com/article/10.3390/diagnostics15080966/s1, Figure S1: ROC curve analysis of ΔCT values. (**a**) Aseptic specimens and (**b**) septic specimens compared with culture results of rare or more than rare colonies; Figure S2: The difference in fungal broad-range PCR results according to bacterial ΔCT values in a septic ocular specimen (*n* = 97); Figure S3: The difference in fungal broad-range PCR results according to bacterial ΔCT values in an aseptic ocular specimen (*n* = 54); Table S1: Results of conventional PCR and real-time PCR; Table S2: Variables affecting the positive concordance between culture and real-time PCR in aseptic specimens; Table S3: Variables affecting the negative concordance between culture and real-time PCR in septic specimens; Table S4: Variables affecting the negative concordance between culture and real-time PCR in aseptic specimens; Table S5: Results of the microbiome analysis.
