# Supplementary material for: Enhanced Detection of Bacterial Ocular Pathogens: A Comparative Study of Broad-Range Real-Time PCR and Conventional Culture Methods
Source: Diagnostics (Basel). 2025 Apr 10;15(8):966. doi: 10.3390/diagnostics15080966 (PMC12025706; doi:10.3390/diagnostics15080966)
Supplement: Supplementary file 1 [file diagnostics-15-00966-s001.zip › Supplemental Material Figure_20250304.pptx]

## Slide 1
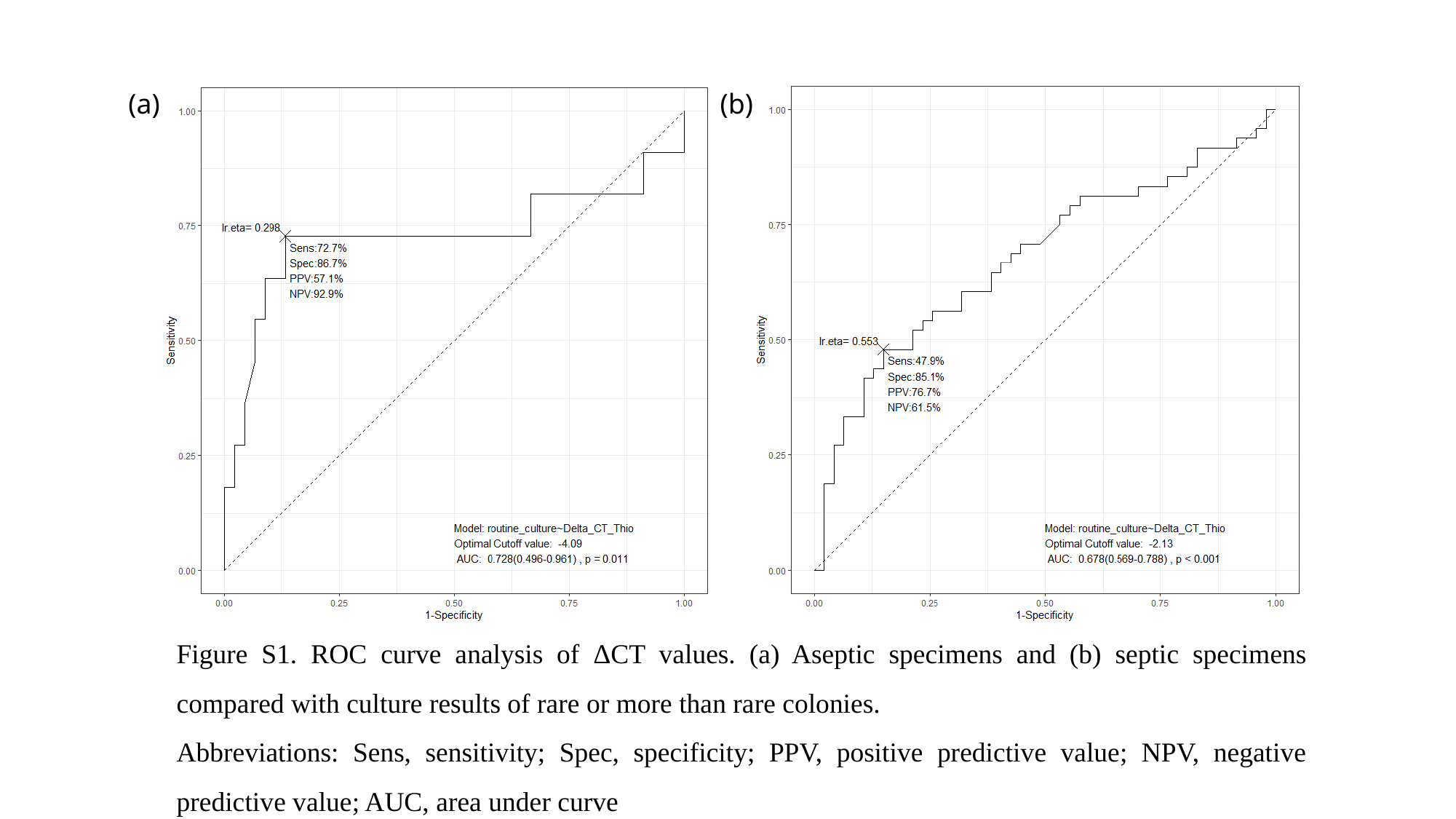

(a)
(b)
Figure S1. ROC curve analysis of ΔCT values. (a) Aseptic specimens and (b) septic specimens compared with culture results of rare or more than rare colonies.
Abbreviations: Sens, sensitivity; Spec, specificity; PPV, positive predictive value; NPV, negative predictive value; AUC, area under curve

## Slide 2
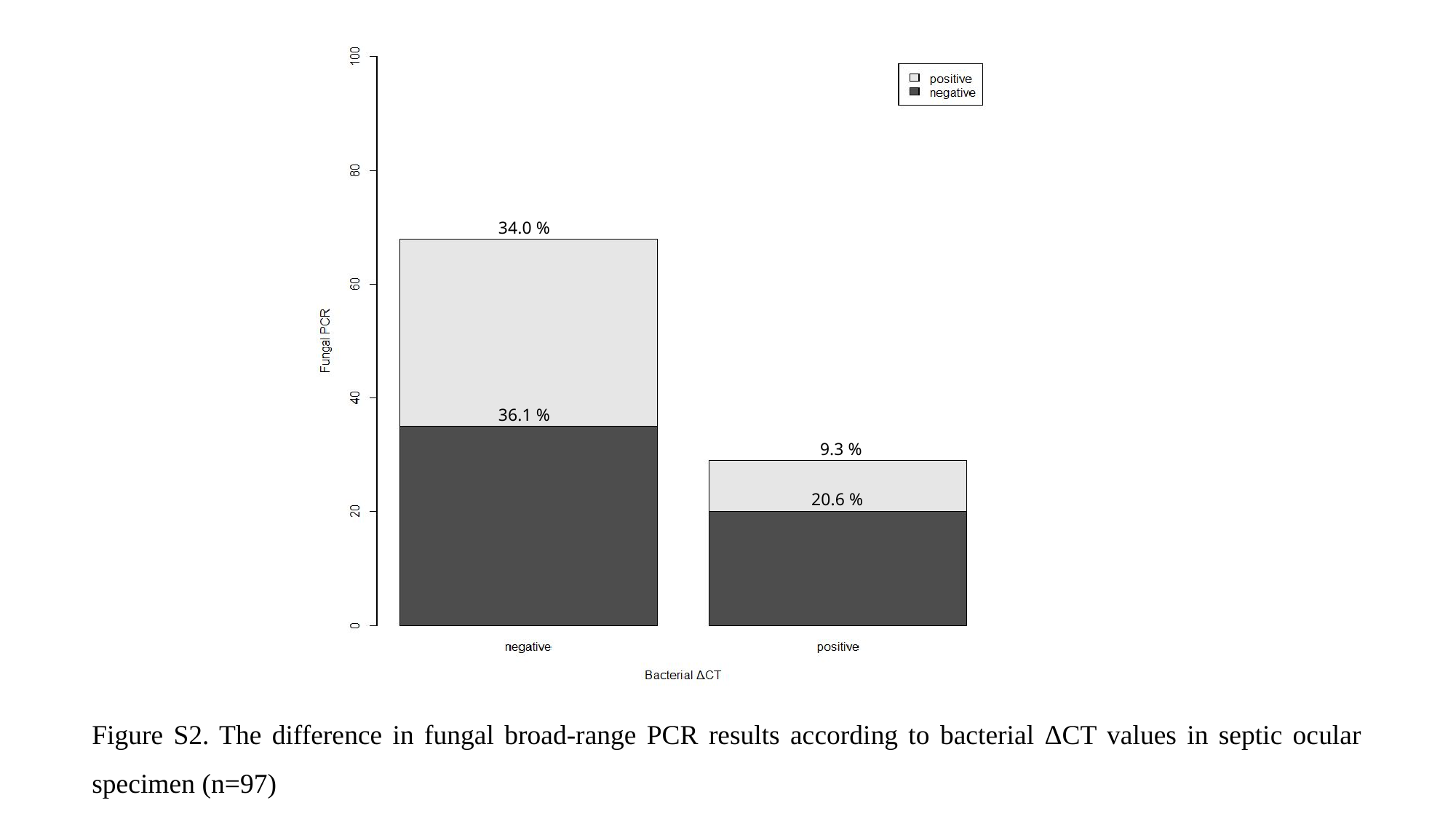

34.0 %
36.1 %
9.3 %
20.6 %
Figure S2. The difference in fungal broad-range PCR results according to bacterial ΔCT values in septic ocular specimen (n=97)

## Slide 3
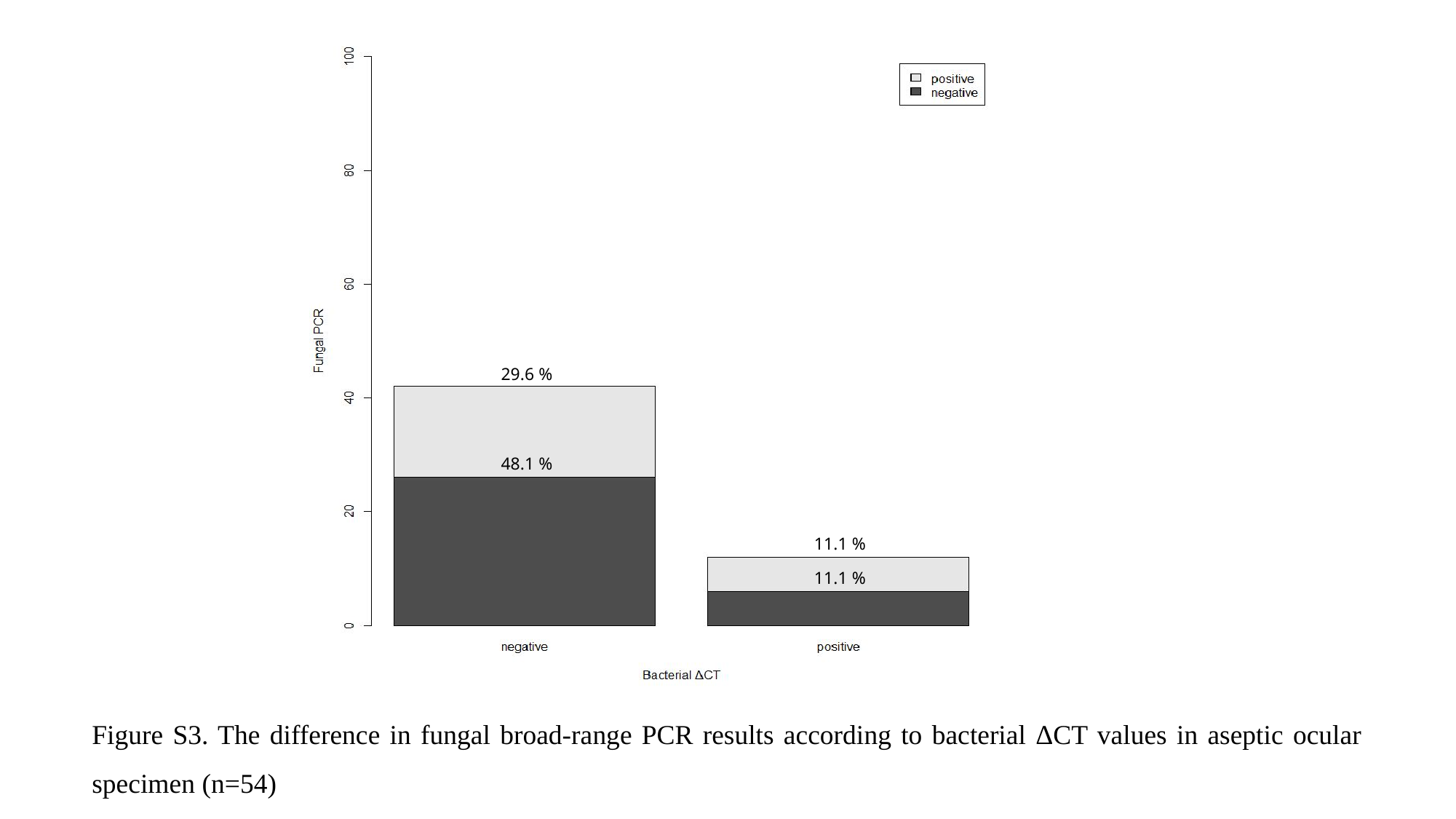

29.6 %
48.1 %
11.1 %
11.1 %
Figure S3. The difference in fungal broad-range PCR results according to bacterial ΔCT values in aseptic ocular specimen (n=54)
